# Relationship between Medical Students' Perceived Stress and Gaming Behavior at King Saud University

**DOI:** 10.1155/2022/3220042

**Published:** 2022-12-01

**Authors:** Turky H. Almigbal, Abdullah A. Alrasheed, Ebtesam S. Almutairi, Rawan A. Alrehaili, Arwa M. Alzahrani, Nourah A. Alhassan, Ranyah Aldekhyyel, Mohammed A. Batais

**Affiliations:** ^1^Department of Family and Community Medicine, College of Medicine, King Saud University, Riyadh, Saudi Arabia; ^2^King Saud University Medical City, King Saud University, Riyadh, Saudi Arabia; ^3^College of Medicine, King Saud University, Riyadh, Saudi Arabia

## Abstract

Gaming addiction has gradually developed among medical students and has been a contentious topic for nearly a decade. This study is aimed at estimating the prevalence of gaming addiction among medical students at King Saud University and examining the relationship between perceived stress levels and gaming addiction. We conducted a cross-sectional study among 370 students from 2019 to 2020 using a self-reporting questionnaire consisting of two validated test scales: the Gaming Disorder Test and the Perceived Stress Scale. The questionnaire was sent to all students through an email. Descriptive analyses and *t*-test statistical tests were used in this study. The prevalence rate of gaming addiction was found to be 4.6%, while the prevalence of perceived stress was confirmed at 95.9%. Students with excellent GPAs and high family income were associated (*p* < 0.001). Younger groups, females, and students with high family incomes showed higher levels of perceived stress than others. According to Pearson's correlation, gaming addiction was not significantly correlated with stress among medical students (*p* > 0.05). According to chi-square test also, no significant association was found between gaming disorder and perceived stress (*χ*^2^ = 4.412; *p* = 0.353). In conclusion, gaming addiction among medical students has scored low prevalence, indicating gaming is not a factor contributing to stress among this group. The high level of perceived stress among medical students should draw attention to the provision of regular psychological care.

## 1. Introduction

The video game has risen to prominence as a kind of gaming among more than 2 billion users across the globe. Addiction to online games can lead to social and/or emotional difficulties when unchecked, excessive, and compulsive [1]. Based on American Society of Addiction Medicine, addiction is defined as to captures both the neurobiological and behavioral etiology of disruption in the brain's mesolimbic reward circuitry and the impact of addictive behaviors [2, 3]. The video game industry is widespread throughout the world, with products running on mobile phones, computers, and video game devices demonstrating positive effects on fundamental mental processes such as perception, attention, memory, and decision-making. Abuse of such a play activity, however, can lead to addiction, i.e., video game addiction [4].

Video game disorder or gaming disorder (GD) is one of the most popular disorders in the globe [5]. Certain people are predisposed to acquiring GD due to their inability to control their gaming behaviors as a form of enjoyment. Individuals with GD account for 0.21-57.5% of the overall population [6]. For the first time, GD was included in the Diagnostic and Statistical Manual of Mental Disorders, Fifth Edition (DSM-5) [7, 8]. A formal diagnosis of GD has been added to the International Classification of Diseases 11th Revision (ICD-11) because of its impact on public mental health [9]. Students have access to a wide range of educational content through various technological tools. Digital games, for example, are rapidly being employed in various learning environments to teach and train students [10]. The World Health Organization (WHO) defines gaming disorder as a pattern of gaming behavior characterized by impaired control over gaming, increasing priority given to gaming over other activities to the extent that gaming takes precedence over other interests and daily activities, and continuation or escalation of gaming despite the occurrence of negative consequences [11, 12]. The prevalence of GD varies by country, as well as by demographics. According to Hawi et al., the prevalence of GD among Lebanese high school students is 9.2% [13]. The incidence of GD was found to be 3.3% by a recent meta-analysis study [14]. Cognitive function, psychopathologies, social connections, and personality characteristics are all risk factors for online gaming addiction.

Many elements contribute popularity of video games, including photorealistic graphics and increasingly sophisticated, complicated, and communicative characteristics. Depression and attention deficit hyperactivity disorder (ADHD) were also risk factors for addiction [15]. Adolescent gaming addiction is frequently accompanied by stress, which has a negative impact on social and familial relationships, self-control and regulation, and academic achievement. There may be a two-way street between gaming addiction and stress. Excessive video game usage has been associated with alterations in the brain's decision-making and emotional control systems, and these changes are connected to stress [16]. Resilience, stress perception, and internet gaming disorder may be linked, and depression could be a mediating factor [17]. Stress is a nonspecific response to pressures placed on the body or distressing events in the surrounding environment. Our perception and response to environmental risks and challenges are more than just a stimulus or a response. In this context, “stressor” refers to anything that might make you feel anxious or tense. [18]. Stress is a fusion of two factors: an individual's incapacity to meet demands or the individual's assessment that they cannot meet those demands. It is common for medical students to be under great stress at both the undergraduate and postgraduate levels [19]. Previous studies conducted in Arab countries, such as Egypt (60%) and Sudan (50%), Lebanese students (62%), and Saudi Arabian medical students (53-72%), demonstrate that medical students in these countries are under a considerable degree of stress [20]. Many studies have been conducted on GD and addictions in medical students in Saudi Arabia [16, 21, 22]. This study examines gaming addiction among King Saud University (KSU) medical students and correlates stress and gaming addiction.

## 2. Material and Methods

### 2.1. Ethics and Other Permissions

Ethical approval (E-19-4440) was sanctioned by an Institutional Review Board (IRB) committee at KSU.

### 2.2. Study Setting

The study was conducted in the College of Medicine at KSU, the capital city of Saudi Arabia. The medical curriculum is a five-year student-centered program incorporating a different approach. Students are introduced to clinical practice anatomy, physiology, pathology, and pharmacology beginning in the first year, with all departments teaching the same system simultaneously, namely the respiratory system. Students must complete system-oriented basic science courses in the first two years, preclinical courses in the third year, and clinical clerkships in the last two years. Medical school's unique teaching and learning methods can be challenging for students, especially during the first year [23]. The inclusion criteria of this study required only KSU medical students in their first to the fifth year of college of medicine to participate. The exclusion criteria were from KSU medical college students or medical students from any other institute.

### 2.3. Sample Size

During our study, there were 1176 registered students for 2019-2020 (1-5^−^year students). A pilot study was already performed with 16 responses to ensure the clarity of the survey tool. Based on a standard sample size equation, we required 290-380 participants to be involved in this study to have a confidence level of 95% and a margin of error of 5%. Based on inclusion and exclusion criteria, we have enrolled 370 questionnaires.

### 2.4. Study Design

In the academic year 2019-2020, we conducted a cross-sectional study that included all first- to fifth-year medical students. We used a self-reporting questionnaire, which consisted of three main sections: (1) demographic information, (2) Internet Gaming Disorder/GD Test (IGD-20), [24] and (3) Perceived Stress Scale (PSS) [25].

Demographic information included age, gender, family income, and grade point average (GPA). The IGD-20 test evaluates video game addiction in the last 12 months through six domains: (1) salience, (2) mood modification, (3) tolerance, (4) withdrawal symptoms, (5) conflict, and (6) relapse. This test consists of 20 statements in multiple choice question (MCQ) format designed to measure the prevalence of gaming addiction on a 5-point Likert scale (1 = strongly disagree, 2 = disagree, 3 = neither agree or disagree, 4 = agree, and 5 = strongly agree). Participants who scored above 71 are considered addicted to playing video games. PSS consists of 10 statements in MCQ format designed to determine the stress levels during the last month on a 5-point Likert scale (0 = never, 1 = almost never, 2 = sometimes, 3 = often, and 4 = very often). Participants who score between 0 and13 have low-stress levels, those between 14 and 26 have moderate stress levels, and participants who score between 27 and 40 have high-stress levels.

We built the survey questions using Google Forms®, and on January 2020, the survey link was distributed to the medical college student council through email. After a month, when low responses were found, we used another method to collect responses (through the WhatsApp student groups each year). Data collection lasted for two months. Students' participation in this study was voluntary.

### 2.5. Statistical Analysis

**Figure 1:**
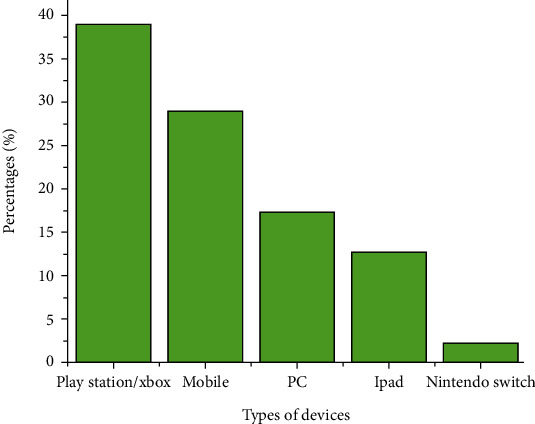
List and prevalence of devices used for playing the video game.

Descriptive statistics were analyzed using mean (M) and standard deviations (SD) for continuous variables, frequencies, and percentages for categorical variables. Categorical variables were used for a single group and for five groups. Student's *t*-test was performed to compare variables between addicted and nonaddicted group. The variables were compared using ANOVA in three groups: low, moderate, and high-perceived stress. Pearson's correlation coefficient was used to find correlation between gaming addiction and perceived stress. Chi-square/Fisher's tests were performed to find an association between gaming addiction (addicted and nonaddicted subjects) and perceived stress levels (Low, moderate, and high).  Figure 1 defines the list of devices used in this study by the students for playing video games. Comparison groups were constructed based on participant demographics, with findings considered statistically significant at *p* < 0.05 [26].

## 3. Results

### 3.1. Characteristics of Participants

In this study, based on sample size estimation, 370 students were finalized and enrolled at KSU medical college. The final response rate was 31.4% after finalizing the study criteria. The complete details of this study's participants are indicated in Table 1. The participating student's age range was between 18 and 25 years. The mean age of all students was 20.97 ± 1.43. The female (53.2%) students were higher when compared to male (47.8%) students in this study participant. The second year (25.9%) actively participated, followed by the first year (25.4%), third year (25.1%), fourth year (14.3%), and fifth year (9.2%). This study's results confirmed that 56.8% of students play video games, and 43.2% do not play video games. The mean age of the students who play video games was 21.00 ± 1.45 years, and those who did not play was 20.91 ± 1.41 years.

### 3.2. List of Devices Used for Playing Video Games

Figure 1 in this study describes 38.9% of students playing video games through play station or Xbox, 28.9% on mobile, 17.3% on PC, 12.7% on iPad, and only 2.2% of the students playing the video games on Nintendo switch.

### 3.3. Video Game Addiction among Students

In this study, Table 2 defines the students' opinions about their habits towards GD. In this study, 25.7% of students agree that they have lost their sleep due to the long session of the video game. About 49% of students disagree about never playing games to feel better. Almost one-third (33.8%) of the students strongly disagree with extending the time limit for gaming sessions. The prevalence factor was 35.2%, as students disagreed that they experience higher irritation when not playing video games. The majority of students, i.e., 31.9% and 29.1%, strongly disagreed with the notion that playing video games leads to developing other hobbies. For this statement, “students could not reduce the gaming time because of very difficult to do it,” 37.6% of the students disagreed. About 31.4% of the students agreed to plan the next video game session when they are not playing. In this study, 40% of students agreed that they play video games to cope with negative feelings. The highest frequency of students who disagreed with increasing their time spent playing the video game was 37.1%. 28.2% of students disagreed, as they feel sad when not playing video games. 42.4% of Saudi students strongly disagree with lying to family members because of their time spent towards GD. Genuinely, 32.4% of students strongly disagreed as they cannot control the stop gaming. Almost 44.3% of Saudi students concluded a strongly disagreed response when asked if GD has become a time-consuming activity in their life. The maximum agreement rate for verifying as students play a video game to avoid bothering them was 36.2% was strongly agreed. The majority of students strongly disagreed (31.9%) that their entire day was insufficient for everything utilized in gaming. Three-quarters of students strongly disagreed that not playing a video game makes them nervous (40.5%). About 33.8% of students strongly disagreed, as gaming has jeopardized their relationship with their partner. Almost 35.2% of students disagreed that restricting their time towards gaming is not possible for them. Most students agreed (31.9%) that gaming has not significantly impacted their daily lives. Almost 32.4% of students disagreed for not in agreement with the notion that their gaming has a detrimental impact on essential aspects of their lives.

### 3.4. Student's Query

Table 3 presents the questions expressed about the students in the previous month. In this table, all participants (*n* = 370) were involved. 42.4% of students confirm that sometimes they may often be upset because something happened unexpectedly in the last month. In the previous month, 34.3% of students confirmed that sometimes they feel as cannot control the important things in their life. Overall, 31.9% of students confirmed it as sometimes. In the last month, 38.1% of students agreed as sometimes as they feel confident in their abilities to handle their issues. Almost 50% of the students in the last month felt as if, sometimes, things were going smoothly their way. In the last 30 days, 41.1% of students sometimes felt they could not cope with everything they had to do. 46.5% was the maximum number of students who could control irritation in their daily life for the past 30 days. In this study, 46.5% of students indicate that sometimes they cannot control irritations in their routine life. Only 46.3% of students, as sometimes in the last 30 days, have confirmed as they were on top of things. Almost 34.1% of the students confirmed as sometimes they become upset due to uncontrolled events in their everyday life for the past month. 35.2% of the students in the last month sometimes felt that difficulties were piling so high that they could not be solved.

### 3.5. Characteristics of Addicted Students towards Their Gaming

Table 4 shows that 17 of the 210 students were addicted to video games, while the remaining 193 were not. The prevalence of gaming addiction was 4.6% (*n* = 17) among the enrolled students. The mean age of addicted students was 21.82, while that of nonaddicted students was 20.96. In an addicted section, 0.05% of students were in their first and final years, while 35.3% were in their second and fourth academic years. Only 17.6% of students are in their third year of study. Nonaddicted students make up 25.9%, 24.9%, 25.4%, 14%, and 9.8% of the student belongs in their respective academic years. Around 47.1% of addicted students had a GPA of 4.00-4.49, 23.5% had a GPA of 3.50-3.99, 17.6% had a GPA of 3.00-3.49, and 11.8% had a GPA of 4.5-5.00. The majority of addicted students (35.3%) have a low monthly family income of 5,000-10,000 SAR, 29.4% have a monthly family income of 10,001-20,000 SAR, 17.6% have a monthly family income of 40,001-50,000 SAR, 11.8% have a monthly family income of >50,000 SAR, and 0.05 have a monthly family income of >50,000 SAR. According to Student's *t*-test, there was a significant difference in GPA and monthly family income of addicted and nonaddicted students (*p* < 0.01).

### 3.6. Perceived Stress and Student's Characteristics

Table 5 in this study showed an association between stress levels and student characteristics. The sample size in this study was 363 rather than 370 due to a lack of insufficient data. 95.9% of students reported significant levels of stress. In this study, 54.3% of female students had the highest levels of stress in comparison to male students, i.e., 45.7%. Both genders showed high-stress levels, but slightly higher among females (54.3%). The moderate level of stress was found to be 30.8%, which is high among third-year students, and high stress levels among first- and second-year students were 25.9% and 25%, respectively. The students with 50.6% have reported significant stress levels had a higher proportion of an outstanding GPAs ranging in between 4.50 and 5.00. In this study, 24.1% of students with high stress levels had a family income between 20,001 and 30,000 SAR and moderate stress levels were revealed in 23.1% of students whose family income ranged between 10,001 and 20,000 and 20,001 and 30,000 SAR. Table 5 shows comparison of variables (ANOVA) between the three groups of low-, moderate-, and high-perceived stress which revealed no significant difference (*p* > 0.05). Pearson's chi-square tests revealed no significant association between gaming addiction and perceived stress (*χ*^2^ = 4.412; *p* = 0.353). The details are found in Table 6.

## 4. Discussion

The purpose of this study was to investigate the addiction role of gaming behavior in KSU Medical City students and to correlate the perceived stress with a video game. The output of this study confirms that 45% of students disagree with the presented questions regarding their gaming addiction (Table 2). In this study survey, 44.3% of students said they strongly disagreed, probably because they view gaming as a serious and time-consuming activity in their daily life; 49% of students strongly disagreed with never playing video games to feel better. 25.7% of students agreed as they lost their sleep due to the long gaming session. In this study, 35.3% of students were addicted to gaming in their second and fourth academic years. The students with 50.6% have reported significant stress levels had a higher proportion of an outstanding GPAs ranging in between 4.50 and 5.00. In this study, 24.1% of students with high stress levels had a family income between 20,001 and 30,000 SAR and moderate stress levels were revealed in 23.1% of students whose family income ranged between 10,001 and 20,000 and 20,001-30,000 SAR. No significant association was observed between gaming addiction and perceived stress (*χ*^2^ = 4.412; *p* = 0.353).

Our study results were in accordance with other global studies, and we confirmed that the prevalence of gaming addiction among our participants was lower than in other studies examining gaming addiction among other study groups [13, 27–32]. In our study, almost all students indicated high levels of stress when compared with the documented previous studies [33–35]. One of the limitations of our study could be the timing, which may have a potential factor in viewing high levels of perceived stress. Another limitation of this study is that we were unable to record the response rate of students.

Our study was carried out throughout the university young doctor's examination time, which could be one of the possible explanations for these results, including study anxiety, insufficient study time, parents' high expectations, and a lack of sleep. It is critical to determine students with low learning or organizational capabilities, those with mental health issues, or those who experience significant personal difficulties, as these groups are more affected by stress. Such students may require extra monitoring, support, and advice, especially during the first years of the medical program. It is also crucial to develop an organized counselling process for such students.

In recognition of the effects of stress on the performance of medical students, several international organizations have recommended the development of specific programs built to recognize students with symptoms of stress and provide more guidance and support during transition periods [36] and provide health promotion programs to deal with problems at an early stage as a preventive measure. These measures have proven to decrease the harmful effects of stress on students' health and academic performance [37].

While our main intention was to determine the prevalence of gaming addiction among medical students, compared to the significant stress levels reported by our subjects, we found a surprisingly low rate of gaming addiction [35]. This may be because the demands of medical school are very high. Students are required to dedicate many hours of studying to succeed in medical school, which leaves students with minimum hours left for other nonacademic related activities, including playing online/video games.

Our research reveals that gender influenced gaming behavior among students who did play online/video games, with males playing more than females, which was consistent with previous research [15, 38–43]. Conventionally, online/video games are perceived as hobbies for the young, especially among males. This extensive use may be due to the need to enhance one's self-esteem, self-identification, and self-image by sharing one's enthusiasm with others, which increases the risk of addiction. While there is evidence that gamers come from a variety of backgrounds, there has been an increase in female gamers in recent years. This increase in female engagement could be attributed to the introduction and availability of games via smartphones and other portable devices.

Our analysis found no significant correlation between gaming addiction and stress among medical students. These findings contrast with studies that found that addiction to gaming makes individuals experience more anxiety and depression [40]. Most (95.4%) of the medical students in our study were not addicted to online/video games, even with perceived high-stress levels. These findings suggest that our participants did not use gaming as a coping strategy to overcome stress, unlike the gaming behaviors of other nonmedical students when experiencing stress [43, 44]. It is worth exploring the reasons contributing to the differences in preferences of stress coping strategies between medical and nonmedical students.

Our study had several limitations, including the relatively small sample size, self-reporting measures to measure gaming addiction, and stress. We did not collect or report the game genres that participants were engaged with. Future research efforts should clinically measure stress levels and their effect on gaming behavior. Examining those types of games played and their genres may also help determine gaming addiction. Studies that determine addiction as a disorder through clinical assessments may show different results. These studies should focus on conducting psychological assessments through live sessions with the subject, interviews with family, friends, and colleagues, and measuring the adverse side effects of playing online/video games the individual may experience.

## 5. Conclusion

In conclusion, medical students had no association between perceived stress levels and gaming addiction. High levels of perceived stress reported by our participants should draw more attention to this critical issue to provide regular psychological care and support to medical students. Healthcare systems and educational institutions must identify stressors and establish appropriate early detection measures. A global awareness campaign, particularly among medical students, is required to increase awareness about the negative impact of gaming addiction, which can have a detrimental effect on education. Future cross-sectional studies are recommended to identify gaps in Saudi and the global population.

## Figures and Tables

**Table 1:** Description of sociodemographic features involved in this study.

Characteristic	Frequency (*n*)	Percentage (%)
*Age group (years)*		
18-20	153	41.4%
21-22	157	42.4%
23-25	60	16.2%
*Gender*		
Female	**197**	**53.2%**
Male	177	47.8%
*Year of study*		
First year	94	25.4%
Second year	96	25.9%
Third year	93	25.1%
Fourth year	53	14.3%
Fifth year	34	9.2%
*GPA*		
<3	02	0.6%
3.00-3.49	10	2.7%
3.50-3.99	48	13.0%
4.00-4.49	126	34.0%
4.50-5.00	184	49.7%
*Family income*		
5000–10,000	34	9.2%
10,001–20,000	69	18.6%
20,001–30,000	88	23.8%
30,001–40,000	54	14.6%
40,001–50,000	42	11.4%
>50,000	83	22.4%
*Do you play video games?*		
Yes	210	56.8%
No	160	43.2%

**Table 2:** Students involved in this study and their addiction with gaming session.

Students' opinion on gaming disorder (*n* = 210)	Strongly disagree	Disagree	Neutral	Agree	Strongly agree
Students often lost their sleep due to the long gaming session	43 (20.5%)	48 (22.9%)	45 (21.4%)	54 (25.7%)	20 (9.5%)
Students never play games in order to feel better	54 (25.7%)	103 (49%)	22 (10.5%)	18 (8.6%)	13 (6.1%)
Students often increase the duration of time for gaming session	71 (33.8%)	66 (31.4%)	31 (14.8%)	28 (13.3%)	14 (6.7%)
Students feel more irritable when not gaming	72 (34.3%)	74 (35.2%)	42 (20%)	18 (8.6%)	04 (1.9%)
Students develop other hobbies because of gaming	67 (31.9%)	61 (29.1%)	32 (15.2%)	39 (18.6%)	11 (5.2%)
Students could not reduce the gaming time but it is very difficult to do it	55 (26.2%)	79 (37.6%)	37 (17.6%)	28 (13.4%)	11 (5.2%)
Students plans for next gaming session when they are not playing	53 (25.2%)	66 (31.4%)	36 (17.2%)	47 (22.4%)	08 (3.8%)
Students play games to cope up with their bad feelings	31 (14.8%)	31 (14.8%)	35 (16.6%)	84 (40%)	29 (13.8%)
Students increase the amount of time they spend playing video games	39 (18.6%)	78 (37.1%)	50 (23.8%)	39 (18.6%)	04 (1.9%)
Students feel sad when they are not able to play the games	50 (23.8%)	59 (28.2%)	45 (21.4%)	45 (21.4%)	11 (5.2%)
Students lie to their family members because of time spent for gaming	89 (42.4%)	67 (31.9%)	23 (10.9%)	25 (11.9%)	06 (2.9%)
Students could not control the stop gaming	68 (32.4%)	63 (30%)	34 (16.2%)	33 (15.7%)	12 (5.7%)
Students believe that gaming has become a more time-consuming activity in their life	93 (44.3%)	55 (26.2%)	20 (9.5%)	28 (13.3%)	14 (6.7%)
Students play game to avoid their bothering	34 (16.2%)	30 (14.3%)	40 (19%)	76 (36.2%)	30 (14.3%)
Students believe that whole day is not enough for everything that is utilized in gaming	67 (31.9%)	59 (28.1%)	36 (17.2%)	32 (15.2%)	16 (7.6%)
Students become nervous when they are unable to play a game for whatever reason	74 (35.2%)	85 (40.5%)	24 (11.4%)	23 (11%)	04 (1.9%)
Students assume that gaming has jeopardized the relationship with their partner	71 (33.8%)	48 (22.8%)	69 (32.9%)	19 (9.1%)	03 (4.1%)
Students frequently want to limit their gaming time, but many are unable to do so	69 (32.9%)	74 (35.2%)	36 (17.2%)	24 (11.4%)	07 (3.3%)
Students conclude that their daily activity has not affected negatively by gaming	27 (12.8%)	38 (18.1%)	32 (15.3%)	67 (31.9%)	46 (21.9%)
Students believe that their gaming has a detrimental impact on important aspects of life	60 (28.5%)	68 (32.4%)	34 (16.2%)	31 (14.8%)	17 (8.1%)

**Table 3:** List of student's queries raised during the last month.

Students' queries regarding their personal issues from the last month (*n* = 370)	Never	Almost never	Sometimes	Fairly often	Very often
How often were students upset because something happened unexpectedly in last month	18 (4.9%)	61 (16.5%)	157 (42.4%)	82 (22.2%)	52 (14%)
How often did students felt that they cannot control the important things in their life in the last month	27 (7.3%)	47 (12.7%)	127 (34.3%)	94 (25.4%)	75 (20.3%)
How often students felt stressed and nervous in the past month	03 (0.8%)	22 (5.9%)	118 (31.9%)	113 (30.6%)	114 (30.8%)
How often did students feel confident in their abilities to handle their personal issues in the last month	13 (3.6%)	47 (12.7%)	141 (38.1%)	123 (33.2%)	46 (12.4%)
How often did students feel that things were going their own way in the last month	13 (3.6%)	71 (19.2%)	184 (49.7%)	78 (21.1%)	24 (6.4%)
How often did students feel that they could not cope up with everything they had to do in the last month	16 (4.3%)	63 (17%)	152 (41.1%)	89 (24.1%)	50 (13.5%)
How often were students able to control irritations in their daily life in the last month	08 (2.2%)	50 (13.5%)	172 (46.5%)	113 (30.6%)	27 (7.2%)
How often did each student feel in the last month that they were on top of things	29 (7.8%)	97 (26.2%)	171 (46.3%)	49 (13.3%)	24 (6.4%)
How often in the last month did students become upset because their things were out of control	24 (6.6%)	74 (20%)	126 (34.1%)	85 (23%)	61 (16.3%)
How often did students feel like difficulties were piling so high that they could not be solved in last month	29 (7.8%)	70 (18.9%)	130 (35.2%)	83 (22.4%)	58 (15.7%)

**Table 4:** Gaming addiction and students' characteristics (*n* = 210).

Characteristic	Addicted^∗^ (*n* = 17)	Not addicted^∗^ (*n* = 193)	*p* value
Mean age (years)	21.82	20.96	0.78
Gender	*n* (%)	*n* (%)	
Female	8 (47.1%)	65 (35.1%)	0.37
Male	9 (52.9%)	120 (64.9%)	
Academic year	*n* (%)	*n* (%)	
First year	1 (5.9%)	49 (26.5%)	0.09
Second year	6 (35.3%)	47 (25.4%)	
Third year	3 (17.6%)	45 (24.3%)	
Fourth year	6 (35.3%)	27 (14.6%)	
Fifth year	1(5.9%)	17 (9.2%)	
GPA	*n* (%)	*n* (%)	
5–4.50	2 (11.8%)	96 (51.9%)	**0.001**
4.49-4	8 (47.1%)	57 (30.8%)	
3.99–3.50	4 (23.5%)	27 (14.6%)	
3.49-3	3 (17.6%)	3 (1.6%)	
<3	0 (0%)	2 (1.1%)	
Monthly family income	*n* (%)	*n* (%)	
5000–10,000	6 (35.3%)	16 (8.6%)	**0.001**
10,001–20,000	5 (29.4%)	23 (12.4%)	
20,001–30,000	1(5.9%)	46 (24.9%)	
30,001–40,000	0 (0%)	26 (14.1%)	
40,001–50,000	3 (17.6%)	20 (10.8%)	
>50,000	2 (11.8%)	54 (29.2%)	

**Table 5:** Perceived stress levels and students' characteristics (*n* = 363).

	Perceived stress levels			*p* value
	Low (*n* = 2)	Moderate (*n* = 13)	High (*n* = 348)	
Mean age (years)				
	21.50	21.46	20.96	
Gender	*n* (%)	*n* (%)	*n* (%)	
Male	1 (50.0%)	9 (69.2%)	159 (45.7%)	0.246
Female	1 (50.0%)	4 (30.8%)	189 (54.3%)	
Academic year	*n* (%)	*n* (%)	*n* (%)	
First year	—	2 (15.4%)	90 (25.9%)	0.214
Second year	1 (50.0%)	1 (7.7%)	90 (25.9%)	
Third year	—	4 (30.8%)	87 (25.0%)	
Fourth year	—	3 (23.1%)	50 (14.4%)	
Fifth year	1 (50.0%)	3 (23.1%)	31 (8.9%)	
GPA	*n* (%)	*n* (%)	*n* (%)	
4.50-5.00	—	4 (30.8%)	176 (50.6%)	0.531
4.00-4.49	2 (100.0%)	7 (53.8%)	114 (32.8%)	
3.50-3.99	—	2 (15.4%)	46 (13.2%)	
3.00-3.49	—	—	10 (2.9%)	
<3	—	—	2 (0.6%)	
Monthly family income	*n* (%)	*n* (%)	*n* (%)	
5000-10,000	—	2 (15.4%)	32 (9.2%)	0.468
10,001-20,000	2 (100.0%)	3 (23.1%)	64 (18.4%)	
20,001-30,000	—	3 (23.1%)	84 (24.1%)	
30,001-40,000	—	2 (15.4%)	50 (14.4%)	
40,001-50,000	—	1 (7.7%)	42 (12.1%)	
>50001	—	2 (15.4%)	76 (21.8%)	

**Table 6:** Chi-square test results for association between gaming addiction and perceived stress.

	Value	Df	*p* value
Pearson chi-square	4.412	4	0.353
Likelihood ratio	4.196	4	0.380

Df: degree of freedom.

## Data Availability

The data used to support the findings of this study are incorporated within this article.
